# Women’s experiences of the OASI Care Bundle; a package of care to reduce severe perineal trauma

**DOI:** 10.1007/s00192-020-04653-2

**Published:** 2021-01-21

**Authors:** Posy Bidwell, Nick Sevdalis, Louise Silverton, James Harris, Ipek Gurol-Urganci, Alexandra Hellyer, Robert Freeman, Jan van der Meulen, Ranee Thakar

**Affiliations:** 1 Centre for Quality Improvement and Clinical Audit, Royal College of Obstetricians and Gynaecologists, 10-18 Union Street, London, SE1 1SZ UK; 2 Health Service and Population Research Department, King’s College London, De Crespingy Park, London, SE5 8AF UK; 3 Royal College of Midwives, 10-18 Union Street, London, SE1 1SZ UK; 4 Chelsea and Westminster Hospital, 369 Fulham Road, London, SW10 9NH UK; 5 Department of Health Services Research and Policy, London School of Hygiene and Tropical Medicine, 15-17 Tavistock Place, London, WC1H 9SH UK; 6 University Hospitals Plymouth NHS Trust, Derriford Road, Devon, PL6 8DH UK; 7 Croydon Health Services NHS Trust, 530 London Road, Croydon, CR7 7YE UK

**Keywords:** ‘Care bundle’, ‘Hands-on’, Maternity, OASI, Perineal trauma, Qualitative

## Abstract

**Introduction and hypothesis:**

Obstetric anal sphincter injury (OASI) is a severe form of perineal trauma that can occur during vaginal birth. Long-term morbidities include anal incontinence and psychosocial disorders. To reduce these injuries within England, Scotland and Wales, the OASI Care Bundle was introduced to 16 maternity units (January 2017–March 2018). The OASI Care Bundle comprises four elements: (1) antenatal information, (2) manual perineal protection, (3) medio-lateral episiotomy (when indicated) and 4) recognition and diagnosis of tears. As part of the project evaluation, a qualitative study was conducted to explore women’s experiences of the OASI Care Bundle.

**Methods:**

Semi-structured interviews were conducted with women (*n* = 19) who received the OASI Care Bundle as part of their maternity care. This was to explore their experience of each element. A thematic analysis of the interview data was performed.

**Results:**

Three themes were identified: (1) memories of touch, whereby women reported that a ‘hands-on’ approach to perineal protection was a positive experience; (2) midwife as a supportive guide, where women reported that good communication facilitated a calm birth and post-birth diagnosis; (3) education: women need more information about perineal trauma.

**Conclusion:**

This study contributes to the literature through its exploration of women’s experiences of perineal protection techniques and diagnosis of perineal trauma. Interviewed women indicated that they did not experience any of the care bundle elements as an intrusion of their physical integrity. Additionally, an urgent need was identified for more information about perineal trauma in terms of risk, prevention and recovery.

## Introduction

In the United Kingdom (UK) an estimated 85% of vaginal births result in some trauma to the genital tract [1]. The majority of these injuries heal well, causing no long-term sequelae. However, obstetric anal sphincter injury (OASI), the collective term for third- and fourth-degree perineal tears, is a severe complication of vaginal birth [2]. The aetiology of OASI is multifaceted and known risk factors include birthweight > 4 kg, primiparity and an instrumental (assisted) vaginal birth [3]. OASI can cause significant long-term physical and psychosocial morbidities including incontinence, chronic pain, sexual dysfunction and post-traumatic stress disorder [4–7]. In the English National Health Service (NHS) reported OASI rates tripled among primiparous women from 1.9% in 2000 to 5.9% in 2011 [8]. Similar trends have been seen in other countries [9–12]. Increased rates have been linked to improved recognition of tears and variations in intrapartum practice, such as sub-optimal episiotomy use and differing approaches to perineal protection [8, 13–15].

Historically, perineal protection was a key priority of midwives, with one of the earliest writings about perineal care provided by Soranus of Ephesus (98–138 AD) who stated that hands should be used to support the perineum together with a compress to restrain the anus [16]. In 1871, the physician Goodell reported disparities in perineal care that included ‘*those who conscientiously use support because “something must be done” and yet others will not touch the perineum on any account’* (Goodell 1871, cited in [17]). Diverse opinions on perineal protection continue today. This is reflected in current UK guidelines [18], which state that clinicians may utilise one of two methods to protect the perineum during crowning of the fetal head. These are:

1. The ‘hands-on’ approach, which recommends pressure applied to the advancing vertex and/or stretching perineum.
2. The ‘hands-poised’ approach, which advocates minimal touch to the perineum.

A recent systematic review on perineal techniques concluded that more research is needed to evaluate which techniques minimise perineal trauma [19]. The authors also note the importance of women’s experiences in order to understand the acceptability of perineal techniques. The OASI Care Bundle Quality Improvement (QI) Project sought to contribute to this understanding as part its comprehensive evaluation. This article reports the qualitative study that was conducted to explore women’s perceptions of each element of the OASI Care Bundle.

### The OASI Care Bundle Quality Improvement Project

**Fig. 1:**
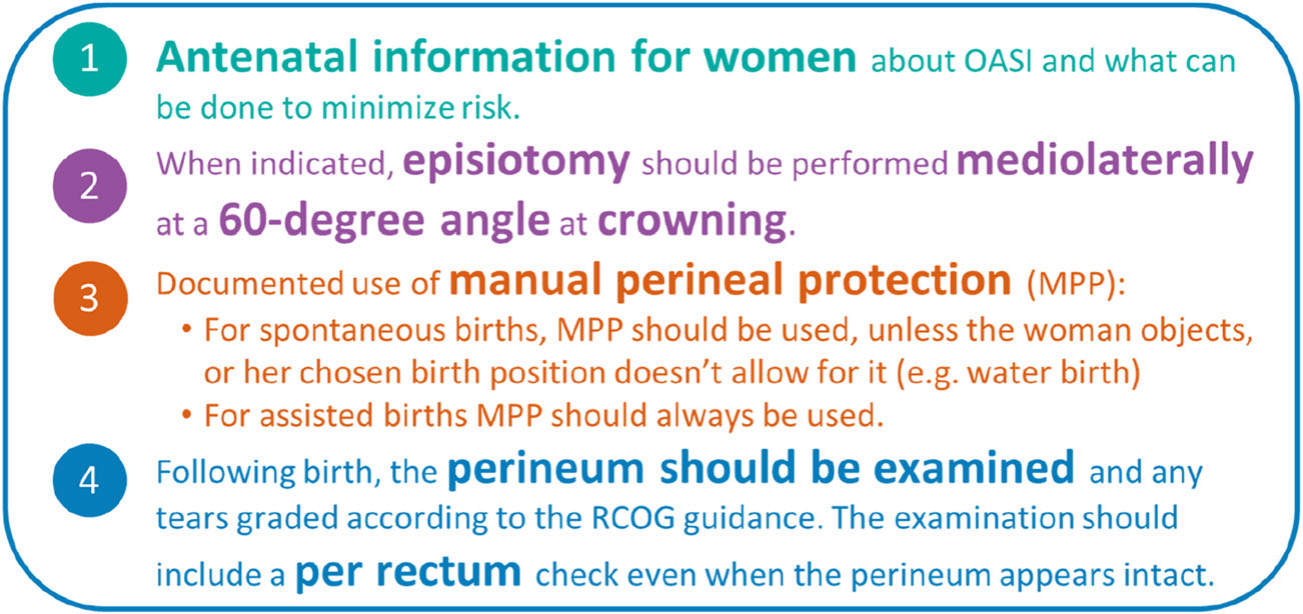
Elements of the OASI Care Bundle

The OASI Care Bundle Quality Improvement (QI) Project was developed to reduce OASI rates within 16 maternity units across England, Scotland and Wales (January 2017–March 2018). Background to the Project and the evaluation methods have been reported in detail [20]. Briefly, the Project involved implementation of the OASI Care Bundle, comprising of four elements (see Fig. 1), supported with an awareness campaign and multidisciplinary training. Implementation of the OASI Care Bundle within each unit was facilitated by local midwives and obstetricians. These clinical ‘champions’ received centralised training at multidisciplinary skills development days. Leadership and support for the Project were provided by two professional bodies, the Royal College of Obstetricians and Gynaecologists (RCOG) and the Royal College of Midwives (RCM).

Good communication between clinicians and women was essential for all four elements of the care bundle, particularly to facilitate a safe and controlled birth. Women were encouraged to be mobile in labour and adopt their chosen birth position [21].

The overall impact of employing the OASI Care Bundle showed a reduction in OASI rates from 3.3% pre-implementation to 3.0% post-implementation (*p* = 0.03), with > 55,000 women included in the analysis [22]. In addition to the clinical impact of the OASI Care Bundle, implementation strategies were evaluated to understand the barriers and enablers towards uptake within units [23].

### Women’s involvement with the OASI Care Bundle QI Project

The Project had women’s involvement throughout inception, implementation and evaluation. In addition, the Project was supported by an Independent Advisory Group, which included user representatives. Component 1 of the OASI Care Bundle (the antenatal information sheet) was co-produced with women’s groups to ensure that the content was appropriate. Skills development days included accounts from women living with the effects of an OASI. On completion of the Project, women and representatives from relevant support organisations were included in events that disseminated the findings.

## Methods

This qualitative study was part of the evaluation process of the OASI Care Bundle QI Project. Enquiry involved a cohort of women who volunteered to participate. Interviews sought to explore women’s perspectives of all four elements of the OASI Care Bundle. As mobility in labour and perceptions of pain were explored in the interviews, it was not possible to include women who had received an epidural or spinal anaesthetic.

Eligibility criteria of participants included:

1. Spontaneous vaginal birth within 1 of the 16 maternity units that took part in the OASI Care Bundle QI Project
2. Nil administration of an epidural or spinal anaesthetic
3. Experience of the OASI Care Bundle

The local clinical champions approached eligible women 6 to 12 hours after childbirth to assess their interest in taking part in the interviews. Women who were interested in taking part gave verbal consent to the clinical champion to pass on their details to the named OASI Researcher, who is also a midwife (PB). Only the woman’s first name and telephone number were required; if any other personal details were provided, these were immediately deleted.

The OASI Researcher contacted the women approximately 6 weeks postpartum to provide more information about the study and to obtain consent. This time frame was considered optimal to allow for recovery after the birth and establishing feeding whilst recall remains good [24]. A proportionate approach to consent was taken, by which if a woman agreed to a time for the interview to be conducted then they had provided their consent to participate. Anonymity was assured and participants were informed that they were free to withdraw at any time.

Interviews took place between June and September 2018. All interviews took place over the phone and lasted 20 minutes on average. A semi-structured interview guide was used to provide a format for the discussion (see Table 1). This was designed to ensure consistency during interviews. The only demographic information collected was parity.

**Table 1:** Summary topic guide for the study

How did your labour start?	
Who did you go to hospital with?	
Did anyone else come?	
What happened when you got to hospital?	
Do you remember the midwife who looked after you?	
What are your memories of the pain that you experienced during labour?	
What was your experience of pain during labour and birth?	
How did you relieve this pain?	
Did you move around during your labour, and if so, how?	
Do you remember what position you were in at the time of birth?	
Was this position your choice, or suggested by your midwife or doctor?	
Do you recall guidance being given to you by your midwife, or doctor, as your baby was being born?	
What did this guidance relate to?	
How did you feel (emotionally, physically) after birth?	
What happened after your baby was born?	
How long did you stay in hospital for?	
Is there anything else you like to add?	

### Analysis

All interviews were audio recorded, except for three where detailed notes were taken whilst the interview was taking place. All audio recordings were transcribed verbatim. All transcripts were anonymised with no personal identifiable markers used. Women were assigned pseudonyms and these have been used for all the quotes.

The six stages of thematic analysis were used to analyse the data [25]. This method allowed a flexible approach to identify core concepts during data collection [26]. Transcripts were read several times and coded by the researcher (PB). NVivo 11 was used to facilitate this process. Ongoing comparison was used to generate themes. Provisional linkages were subsequently developed to link concepts and thus generate theories to enhance our understanding about this cohort of women who received the OASI Care Bundle as part of their maternity care. Data saturation, whereby new themes emerged, was reached after 15 interviews. A further four interviews were conducted to ensure that no new themes developed and theme stability had been reached.

### Ethics review

This study was reviewed as part of the wider OASI Care Bundle QI Project by the NHS Health Research Authority in October 2016 and approved as a service evaluation (Ref 60/86/81). Local approvals were obtained from all the NHS trusts involved in the OASI Care Bundle QI Project. Verbal consent was obtained from women who took part in the interviews.

## Results

In total 19 women who received the OASI Care Bundle as part of their maternity care were interviewed on average 6 weeks’ postpartum. All women had a spontaneous vaginal birth. Obstetric characteristics of the women are found in Table 2 and include multiparity (*n* = 12), nil perineal trauma (*n* = 7), first-degree perineal tear (*n* = 2), second-degree tear (*n* = 7) and a third-degree tear (*n* = 1).

**Table 2:** Characteristics of women who participated in the study

Participant pseudonym	Region	Parity	Labour onset	Perineal trauma experienced
Camilla	1	Multiparous	Induction	None
Olive	1	Multiparous	Induction	None
Paula	1	Multiparous	Spontaneous	None
Valeria	1	Multiparous	Spontaneous	Episiotomy
Priya	2	Multiparous	Spontaneous	None
Sara	2	Multiparous	Spontaneous	Second-degree tear
Khadijah	2	Multiparous	Spontaneous	Second-degree tear
Ciara	2	Primiparous	Induction	Second-degree tear
Naomi	2	Primiparous	Spontaneous	Second-degree tear
Sinead	3	Primiparous	Spontaneous	Second-degree tear
Joanne	3	Primiparous	Spontaneous	Third-degree tear (3a)
Liberty	4	Multiparous	Spontaneous	First-degree tear
Leah	4	Multiparous	Spontaneous	None
Claudia	4	Multiparous	Induction	None
Sue	4	Multiparous	Spontaneous	None
Rose	4	Primiparous	Spontaneous	First-degree tear
Sadie	4	Primiparous	Spontaneous	Second-degree tear
Caitlin	4	Multiparous	Spontaneous	Second-degree tear
Sophie	4	Primiparous	Spontaneous	Episiotomy

### Identified themes

**Fig. 2:**
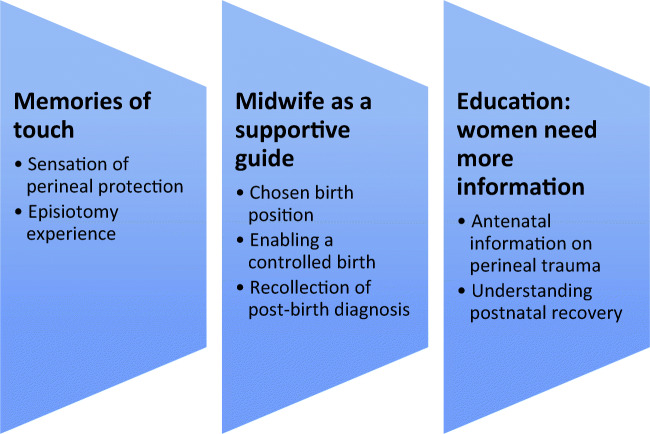
Summary of emergent themes regarding women’s experiences of the OASI Care Bundle

After analysis three themes were identified (see Fig. 2): (1) memories of touch; (2) midwife as a supportive guide; (3) education: women need more information.

1. **Memories of touch**

Two sub-themes were generated from this theme that included (a) sensation of perineal protection and (b) episiotomy experience.

### Sub-theme: Sensation of perineal protection

The majority of women (*n* = 14) reported positive memories of the sensation of perineal protection. They used words such as ‘helpful’ and ‘supportive’. No women reported it was a negative experience. Comments included:

‘…Yes, the midwife put her hands and it was really helpful….in that place. It was so comfortable…I don’t know what she did, but a couple of things. She stretched the muscles and it was able to rest. It was good, to me.’ (Khadijah, multipara)

Five women were unable to remember feeling the midwife’s hands on their perineum. They reported that the sensation of their baby being born and pain dominated their memories. Two women felt that the analgesia they had used had altered their perceptions and they felt as if they were in a ‘different world’ or ‘really wiped out’.

‘…Oh, I don’t remember, because I was in the pain, so I don’t remember thinking about the midwife’s hands.’ (Sara, multipara)

‘…I can’t remember so I wouldn’t like to say whether she was holding it in place or not, I can’t remember.…I did not see. I did not look. I was in my own pain.’ (Liberty, multipara)

### Sub-theme: Episiotomy experience

Two women received an episiotomy in this birth. One woman did not show any concern about her episiotomy and recalled that her midwife had good communication skills. Her comments included:

‘…Yes, they had to cut me, I forgot….She was talking me through it. She did really, really well, the midwife. She was very good, this one.’ (Valerie, multipara)

Sophie reported that she did not remember much about having an episiotomy, but attributes this to the fact that she was very tired and the effects of diamorphine. In her words, ‘*I was so tired…my memory is very vague. The midwives were fantastic…they gave us lots of support, my husband told me later.’* Sophie also reported that she had been made aware of episiotomies during antenatal classes. Her comments included:

‘…A midwife talked about what could happen during the delivery…. I knew I might get cut, so that’s why I did a lot of exercise, trying to avoid that….I only found out I was cut after I had a baby, not before, because I really can’t remember. I was out most of the time.’ (Sophie, primipara)

(2) **Midwife as a supportive guide**

This theme revealed three sub-themes that included: (1) chosen birth position, (2) enabling a controlled birth and (3) recollections of post-birth diagnosis.

### Sub-theme: Chosen birth position

The majority of women (*n* = 16) reported that they chose the position that they gave birth in. Chosen positions included all-fours, semi-recumbent and left lateral. Comments included:

’…Yes, I kept changing round’. I was on my knees sat upright on the bed. I kept turning round…I was kind of laying on my right side on my back as his head came.’ (Sadie, primipara)

A few women (*n* = 3) recalled that their midwife had suggested their birth position, because they needed additional guidance due to rapid or imminent birth. Comments included:

‘…I couldn’t stand any more I was tired…so she lowered the bed and I got on it…she said that baby was going to come very quickly.’ (Olive, multipara)

### Sub-theme: Enabling a controlled birth

The majority of women (*n* = 17) reported that their midwife had communicated clearly with them to enable a slow and controlled birth. Comments include:

‘…The midwife had explained everything that she was going to do….I listen to the midwife as I know without them I am not going to get anywhere….She was talking me through—it was fab….I couldn’t believe that I didn’t need stitches.’ (Sue, multipara)

‘…The midwife knew what she was doing. It helped. I was told to slow breath and push bit by bit.’ (Paula, multipara)

Two women reported that they had a rapid labour and could not remember the midwife communicating with them. These women spoke of ‘panic’ and birth being ‘too quick’ as opposed to being slow and controlled. Comments included:

‘…It was so quick….I wanted an epidural as I have a low pain threshold, but there wasn’t time….it was too quick….I don’t remember what the midwife was doing.’ (Joanne, primipara)

‘…I didn’t listen at the time to the midwife because I was in so much pain! Panic, in pain and panic….it was so quick….it was like that with my first baby.’ (Sara, multipara)

### Sub-theme: Recollection of post-birth diagnosis

When women were asked if they had a rectal examination following birth (as part of a systematic assessment to recognise and diagnose any perineal trauma) a few (*n* = 5) did not remember this examination. One woman reported that she had to go to the operating theatre with a retained placenta, so this was the focus post-birth. Comments included:

‘…I think they were trying to see if the placenta would come out because I’ve always bled as well….had haemorrhage afterwards. To be honest, I think we were probably more focussed on that than anything else. Well, I was anyway. I don’t know what the midwife was doing!’ (Priya, multipara)

‘…I really don’t remember that. I genuinely can’t remember.’ (Naomi, primipara)

Interestingly, one woman who could remember feeling the midwife’s hands performing perineal protection (see ‘Memories of touch’) could not remember the rectal examination. Her comments included:

‘…I don’t remember. But she probably must have done, but in the midst of everything, I had baby and so I was quite happy. So yes, I’m sure.… but now I can’t really remember everything.’ (Olive, multipara)

Most women (*n* = 14) reported that they remembered receiving information from their midwife about the risks and benefits that are associated with a rectal examination. All these women reported that they understood for this rationale for this examination and were unconcerned by it. Women reported that the rectal examination was an acceptable way to diagnosis any trauma as they wanted to be reassured that there are no underlying problems. Comments included:

‘…I suppose I wanted to make sure that everything was okay, but it wasn’t very comfortable.’ (Claudia, multipara)

‘...She explained it to me and she said she will do it. Maybe she said, ‘It might be uncomfortable.’ I can’t remember, but she was very nice about it.’ (Liberty, multipara)

‘…She asked me if I was happy with that and she explained before she went ahead and did it.’ (Ciara, primipara)

(3) **Education: women need more information**

This theme revealed two sub-themes that included: (1) antenatal information on perineal trauma and (2) understanding postnatal recovery.

### Sub-theme: Antenatal information on perineal trauma

Women were asked whether they remembered receiving a copy of the information sheet about the OASI Care Bundle during pregnancy. The leaflet is provided in Appendix 1. The majority of women (*n* = 15) reported that they did not remember receiving this leaflet. Comments included:

‘…To be honest, I’ve been given loads of stuff. I don’t really look at them. Unless it’s something that’s got, ‘Important,’ or something on it…I honestly couldn’t say for sure about that one. I’m afraid it just goes in the bin.’ (Priya, multipara)

‘…I can’t remember, because I got so much paperwork. I got leaflets everywhere I went, so I’m not actually sure.’ (Sinead, primipara)

One woman remembered being given the leaflet during labour; however, she had limited recall about the information that was provided within it. Her comments included:

‘…I was in labour at the time, I don’t remember. I just remember it was a good useful leaflet and they helped me very well.’ (Caitlin, multipara)

This highlighted an apparent lack of information about perineal trauma provided by healthcare professionals to women during the antenatal period. Women felt that it was up to them to find out information from other sources, such as friends or the internet. Comments included:

‘…You get leaflets about vaccinations and stuff. It was just the only information I got really was what I’d looked up online. I’d been to like a pregnancy yoga class and…a lot of women there had suggested the oil and the massaging there. It was just finding my own information really.’ (Sadie, primipara)

In addition, women felt that even if perineal trauma was talked about during pregnancy, these conversations did not extend to information about the severity of injuries that could potentially be sustained. Comments included:

‘…It is hard to comprehend that [anal sphincter] is where a tear could be.’ (Joanne, primipara)

Women acknowledged that information about perineal trauma might be worrying, especially for those who might be anxious; however, they firmly felt that information should be freely available and it was up to the women themselves to see how much they accessed. Comments included:

‘…I mean it’s all frightening, the whole thing is frightening but it’s just one of those things we’ve got to know about.’ (Caitlin, multipara)

‘…It depends what kind of personality you’ve got. I like to know things in advance because I like to prepare myself for the worst, but also is there anything I can do about it? I suppose if you’re a person that hates anything bloody or scary, medical then perhaps you wouldn’t like it. I, myself, would be quite happy to have more information about it, if only I’d known when I had my first.’ (Priya, multipara)

### Sub-theme: Understanding postnatal recovery

All women (*n* = 19) reported that there is a lack of information about postnatal recovery from perineal trauma. Comments included:

‘…I don’t think people know enough about it generally. I don’t think men know about it. I think it comes as a bit of a surprise to them that there are suddenly stitches, and they aren’t aware of that….people don’t appreciate that you are recovering from lack of sleep, you are sore, you’ve been stitched up. It isn’t really talked about.’ (Naomi, primipara)

‘…Just to understand why the tearing happens and what happens when it heals. No one really tells you whether anything goes back to normal; you just kind of have to wait and see!’ (Sadie, primipara)

Furthermore, very few women reported that they had a postnatal perineal check from a midwife. For example, when one woman was asked if the community midwife had checked perineum during a home visit she said:

‘…No and I was shocked. The community midwife said, ‘No, we don’t do that.’ I said, ‘In the past they always did.’ (Liberty, multipara)

## Discussion

This study presents insight into women’s experiences of perineal care during vaginal birth. Our findings suggest that the four elements of the OASI Care Bundle are acceptable to women. Our findings also highlight that women receive insufficient information about perineal trauma, both during pregnancy and postpartum. Previous studies to reduce severe perineal trauma have focussed on the effectiveness of interventions [14, 27–29] rather than women’s experiences. It is essential that women’s voices and experiences are understood throughout all aspects of maternity care.

Reflection on the themes provides an opportunity to understand how the experiences of women in this study compare with the existing literature—we offer this reflection below.

### Memories of touch

Women reported that a ‘hands-on’ approach to perineal protection was a positive and supportive experience. In a phenomenological study, a midwife reported that she did not want to touch the perineum as she believed it was painful for women, though the premise for this belief is not clear [30]. Non-adoption of a ‘hands-on’ approach appears to be largely driven by midwives, perhaps partly because of misinterpretation of the HOOP trial (Hands On Hands Poised) or an opposition to a medicalised, intervention-heavy approach [31, 32]. Although our findings suggest that a ‘hands-on’ approach is acceptable to women additional research is required to assist unresolved clinical practice issues regarding this practice and optimise perineal outcomes.

Episiotomies have diverse meanings to women depending on social context, professional background and personal perspective [33]. This means that they can have a wide range of physical and psychological consequences [34]. Women in this study did not perceive their episiotomy as a negative experience and felt it had been necessary to expedite a safe birth. This finding is similar to recent research which found that despite the painful aspect of episiotomies, women would be willing to have the procedure again if it was a safety requirement [35]. Women in this study appreciated the optimal communication from their midwives as a means of understanding their maternity care. One study reports that episiotomies have been performed without women’s consent and understanding [34] and one woman in this study reported no memory of the episiotomy being performed. It is, however, unclear as to what effect analgesia, such as diamorphine, can have on memories of intrapartum events. Some research suggests that analgesia can cause varying degrees of amnesia, which adversely affects birth memories [36].

### Midwife as a supportive guide

The women in this study reported that they had an open and empathetic relationship with their midwives. This relationship facilitated positive birth outcomes. This is supported by research that states that ‘participation-mutuality’ is a central concept of midwifery care [37].

All women used a variety of birth positions and reported that mobility during labour was encouraged. Research suggests that women’s choice of birth position results in better outcomes [38]. However, in terms of perineal outcomes a systematic review found that there was no clear difference in chosen birth position and the incidence of third- or fourth-degree perineal tears [39]. A population study in Sweden has subsequently found that both lithotomy and squatting position result in an increased risk of OASI among nulliparous and multiparous women [40].

Evidence suggests that a calm, controlled birth can reduce perineal trauma [31, 41]. The majority of women in this study reported that they had a calm and controlled birth. Findings from a qualitative exploration of perineal techniques observed that optimal outcomes were a consequence of trust and support, as this empowers women and reduces fear [42].

Enquiry into clinician’s experiences of post-birth rectal examinations found that some were uncomfortable about performing ‘invasive’ procedures [23]. However, the women in this study reported minimal objections and stated that they would rather be examined to ensure there was no underlying damage.

In accordance, research demonstrates that systematic examination of the perineum after birth should involve assessment of the anal sphincter to diagnosis the presence of an OASI. If this does not occur, OASI may be obscured by an apparently intact perineum [43]. Worryingly, women with missed OASIs are more common than expected and can suffer consequences of anal incontinence and unidentified damage may result in a rectovaginal fistula [44]. New guidance and operative proformas can significantly increase the detection rate of OASI to avoid the incidence of undiagnosed trauma [45], which is a breach of duty and may result in legal proceedings [46].

### Education

A major finding from our study was that women receive insufficient information about perineal trauma and postnatal recovery was poorly understood. Comments made are supported by qualitative research into women’s experiences of sustaining in OASI, where participants reported ‘*Nobody warned me about this’* [47]. A core component of the OASI Care Bundle was that pregnant women were given a specially designed leaflet about perineal trauma. Very few women recalled seeing this leaflet. The reasons for this are unclear, although women’s reported limited memory of what information they had been given in pregnancy and overwhelming birth events may be a contributing factor. Findings were similar to those from an Australian study that observed a third of women did not recall receiving advice about diet as part of routine antenatal care [48]. Our results highlight the urgent need for optimal dissemination of reliable, current and comprehensive education materials about perineal trauma. Notably, this initiative should enable women to make informed about her maternity care, as stipulated by the landmark Montgomery v Lanarkshire case of March 2015 [49]. Currently women report that some maternity environments provide negligible information on the risk factors of vaginal birth and this precludes participation in decision making about their care [50].

### Limitations

This study has limitations. The small sample size limited the attainment of data on different forms of perineal trauma. All interviews were conducted on the phone, thus impeding detection of visual cues and establishment of rapport that is typically optimised during face-to-face interviews [51]. The sampling framework for the study contains two levels of selection bias—first, the use of the local clinical champions to initially approach participants; second, all participants were volunteers. The interview questions omitted pertinent questions regarding other methods of perineal protection, such as warm compresses. Interviews were conducted approximately 6 weeks postpartum, so recall bias is possible. Even so, research notes that recall following birth remains high for a long period of time [24]. Fidelity to the OASI Care Bundle was not measured [22]; so, it is unclear whether the conditions and circumstances of this study were implemented in the same manner for all women. Given that the majority of women were multiparous, it is possible that their previous birth experience may have affected their perception of this birth. However, previous birth experience also allowed reflection and for comparisons to be made between the care that they received.

### Strengths

A strength was that women were encouraged to speak freely and provide insight into their own personal experiences. Interviews were conducted by someone who was not associated with their care team which avoided women facing obstacles that prevented them from objectifying problematic issues from those who had provided their care. Women who took part had given birth a range of study units, which had different characteristics and varied clinical contexts. Interviews were conducted by the same researcher, who was also a midwife with adept clinical experience and thus enhanced the consistency of data collection regarding interview content. Findings from this study are likely to be relevant to other women who given birth in the UK.

## Conclusions

This study contributes to the literature on women’s experiences of perineal protection techniques and diagnosis of any trauma that is sustained. Our findings suggest that a ‘hands-on’ approach can provide positive support. Furthermore, examinations post-birth to diagnose trauma are acceptable to women. Postnatal recovery is optimised by the health literary process that precludes maternity distress from unexpected and unexplained perineal injuries. To do this, there is an urgent need to ensure that women are fully informed about the risks of perineal trauma and how to reduce incidence, whilst taking into account individual needs, expectations and circumstances. A follow-on study, OASI2 (2020–2020), will further explore women’s experiences of all elements of the OASI Care Bundle (https://www.health.org.uk/funding-and-partnerships/programmes/oasi2-care-bundle).
